# Artificial Intelligence for Modeling Real Estate Price Using Call Detail Records and Hybrid Machine Learning Approach

**DOI:** 10.3390/e22121421

**Published:** 2020-12-16

**Authors:** Gergo Pinter, Amir Mosavi, Imre Felde

**Affiliations:** 1 John von Neumann Faculty of Informatics, Obuda University, 1034 Budapest, Hungary; pinter.gergo@nik.uni-obuda.hu (G.P.); felde@uni-obuda.hu (I.F.); 2 School of Economics and Business, Norwegian University of Life Sciences, 1430 Ås, Norway; 3 School of the Built Environment, Oxford Brookes University, Oxford OX3 0BP, UK

**Keywords:** call detail records, machine learning, artificial intelligence, real estate price, cellular network, smart cities, telecommunications, 5G, computational science, IoT, urban development

## Abstract

Advancement of accurate models for predicting real estate price is of utmost importance for urban development and several critical economic functions. Due to the significant uncertainties and dynamic variables, modeling real estate has been studied as complex systems. In this study, a novel machine learning method is proposed to tackle real estate modeling complexity. Call detail records (CDR) provides excellent opportunities for in-depth investigation of the mobility characterization. This study explores the CDR potential for predicting the real estate price with the aid of artificial intelligence (AI). Several essential mobility entropy factors, including dweller entropy, dweller gyration, workers’ entropy, worker gyration, dwellers’ work distance, and workers’ home distance, are used as input variables. The prediction model is developed using the machine learning method of multi-layered perceptron (MLP) trained with the evolutionary algorithm of particle swarm optimization (PSO). Model performance is evaluated using mean square error (MSE), sustainability index (SI), and Willmott’s index (WI). The proposed model showed promising results revealing that the workers’ entropy and the dwellers’ work distances directly influence the real estate price. However, the dweller gyration, dweller entropy, workers’ gyration, and the workers’ home had a minimum effect on the price. Furthermore, it is shown that the flow of activities and entropy of mobility are often associated with the regions with lower real estate prices.

## 1. Introduction

Delivering insight into the housing markets plays a significant role in the establishment of real estate policies and mastering real estate knowledge [1,2,3]. Thus, the advancement of accurate models for predicting real estate prices is of utmost importance for several essential economic key functions, for example, banking, insurance, and urban development [4,5,6]. Due to the significant uncertainties and dynamic variables, modeling real estate has been studied as complex systems [7]. The call detail record (CDR) data has recently become popular to study social behavior patterns, including mobility [8,9,10]. The expansion of the new generation technology standard for broadband cellular networks has further increased this data source’s popularity worldwide [11]. Although the literature includes a wide range of applications of CDR from urban planning to land management and from tourism to epidemiology, the CDR’s true potentials in modeling complex systems are still at the very early stage [12]. Consequently, this study explores the potential of CDRs in modeling and predicting the real estate price [13,14,15].

With the development of modern cellular network technologies, the quality of call detail record (CDR) data has been improved continuously [16]. The CDR data characteristics include informative mobility data, for example, travel speed, travel time, and other flow characteristics [17,18,19,20,21,22]. CDR data has great potential to provide insight into mobility and human development in the modern urbanization era [23,24,25,26]. During the past decade, several promising spatial-temporal forecasting models based on CDR data have shown promising results and bright perspectives [27,28].

Data-driven methods and advanced statistical techniques are used worldwide in diverse applications for CDR data [29]. Crowd estimation, mobility pattern modeling, anomaly detection and traffic prediction, profile-based location estimation, mental health and well-being modeling, smart tourism service visualization, poverty prediction, and mapping, identifying significant places, and transport modeling are the successful examples of the application domains for CDR data with promising results [14,15,16]. However, the application of machine learning for CDR data as an emerging technology has been minimal [17,18,19]. Nevertheless, such novel models have shown promising results in developing predictive models with higher accuracy. Artificial neural networks for modeling the trust, K-means clustering for outbreaks modeling, and traffic density analysis [20,21,22,23,24] have shown the potential of machine learning in analyzing the CDR data.

Although CDR data is becoming popular in a wide range of domains at a fast pace, its application in modeling the real estate price is yet to be explored [24,25,26]. Prediction of the real estate based on the CDR data will be significant and beneficial for urban planning, investment, tourism, insurance, security, et cetera [27,28,29]. The present study aims to fill this gap by proposing the hybrid machine learning model of MLP-PSO, which is a multi-layered perceptron (MLP) trained with particle swarm optimization (PSO) to predict the real estate price based on CDR data of the city of Budapest. As artificial neural networks (ANN)-based methods in modeling the CDR data have not been fully explored, the MLP has been proposed in this study. Besides, through using PSO, the parameters are further tuned to achieve the highest performance. The manuscript brings novelty and promising results in modeling CDR with machine learning. Although MLP is a well-known machine learning method, its application in modeling the real estate price based on CDR had not been explored. It is expected that the proposed model can be used in the real-life applications of predicting real estate prices in other cities based on the local CDR data.

## 2. Materials and Methods

### 2.1. Data

The call detail record (CDR) [30,31,32] has recently become popular to study social behavior patterns, including mobility. The expansion of the new generation technology standard for broadband cellular networks has further increased this data source’s popularity worldwide. The true potentials of CDR data in modeling complex systems are still at the very early stage.

In this study, the CDR data has been produced at the Vodafone facilities located in Budapest, Hungary. The spatiotemporal dataset consists of anonymous billing records of calls, text messages, and internet data transfer without specifying the activity type. Thus, a record includes a timestamp, a device ID, and a cell ID. The locations of the cell centroid are also available for geographic mapping. Worth mentioning is that the data accuracy depends on the size of the cells [33,34]. The size of the cells which are located downtown are smaller and placed more densely than in the underpopulated areas. In this study, the data acquisition covers the entire city during spring 2018. This contains 955,035,169 activity records from 1,629,275 SIM cards. However, many of these SIM cards have only a very few activities. Less than 400 thousand SIM cards have regular enough daily activities. Several mobility metrics are calculated using active SIM cards, including the radius of gyration [35] and the entropy [36]. The home and work location are estimated, and the distance of the two locations is also used as a metric. SIM card-based mobility metrics are aggregated to cells based on the subscribers who live or work in a given cell. This results in the following columns used as independent variables for the hybrid machine learning model: dweller entropy, dweller gyration, worker entropy, worker gyration, dwellers’ work distance, and workers’ home distances. Additionally, the dependent variable is the real estate price.

**Figure 1:**
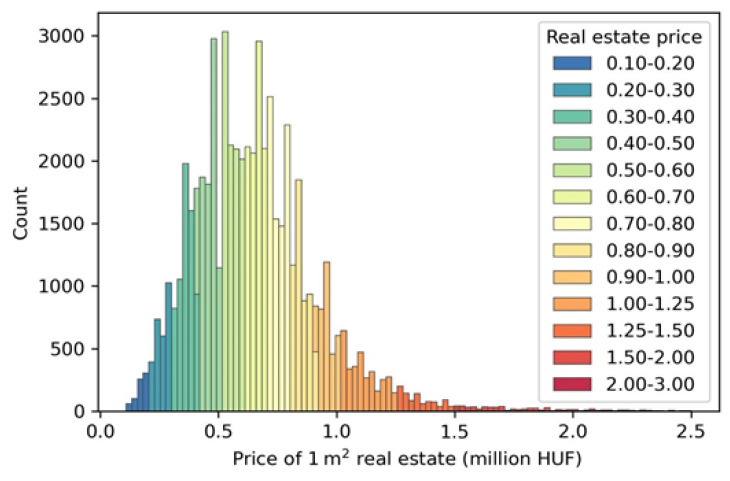
The real estate price histogram.

The normalized real estate price values are associated with every subscriber of the CDR data based on the assumed home location to describe social-economic status. The real estate price data is provided by the ingatlan.com website based on the advertisements in August 2018. The data contains slightly more than 60 thousand estate locations with floor spaces and selling prices (Figure 1 shows its distribution).

**Figure 2:**
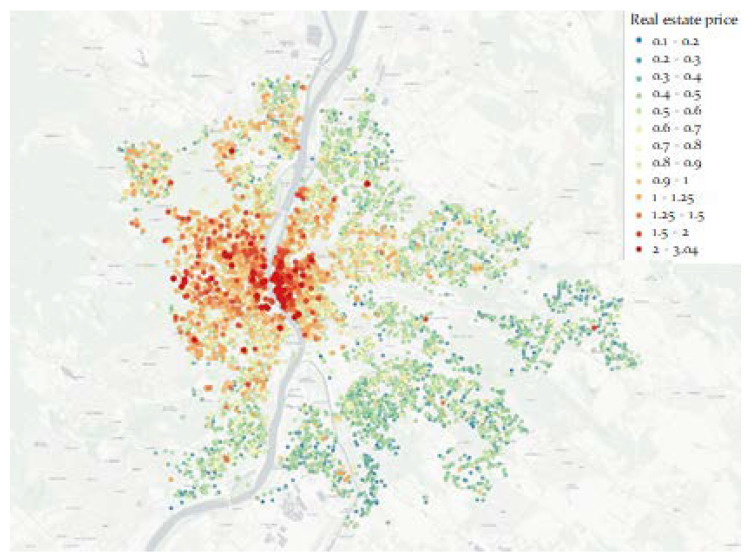
The real estate prices based on the advertisements.

The normalization is performed by dividing the floor space by the selling price. Figure 2 shows the estate advertisements over the map of Budapest. The more expensive estates are represented not just by color but by larger markers as well.

For modeling purposes, the CDR dataset contains mobility entropy data including dweller entropy, dweller gyration, worker entropy, worker gyration, dwellers’ work distances, and workers’ home distances as independent variables for the prediction of estate price as the only dependent variable. Further definitions of the input and output variables are given as follows:

- Norm price: normalized real estate price;
- Dweller entropy: mean entropy of the devices whose home is the given cell;
- Dweller gyration: mean gyration of the devices whose home is the given cell;
- Worker entropy: mean entropy of the devices whose workplace is the given cell;
- Worker gyration: mean gyration of the devices whose workplace is the given cell;
- Dwellers’ home distance: average work-home distance of the devices whose home cell is the given cell;
- Workers’ work distance: average work-home distance of the devices whose work cell is the given cell.

### 2.2. Methods

**Figure 3:**
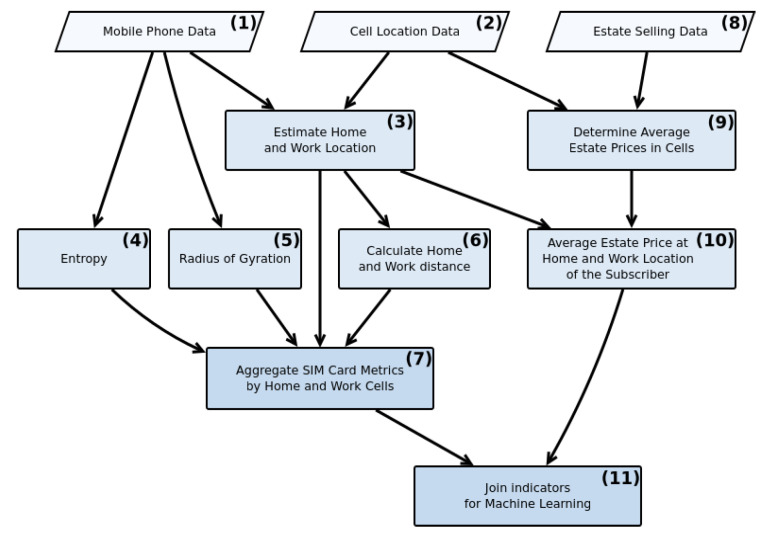
Data preprocessing workflow.

The proposed methodology includes three principal sections, namely, data preprocessing, normalization, and machine learning modeling. The raw CDR data passes through a series of functions to be prepared for the modeling section. Figure 3 represents a simplified workflow of the essential data preprocessing section. According to Figure 3, data preprocessing can be divided into eleven building blocks. After cleaning the input data, the home and work locations have been determined (building block 3) using the most frequent location during and out of the work hours. Then the home-work distance (building block 6), the entropy (building block 4), and the radius of gyration (building block 5) are calculated for every SIM card. Using the market selling prices, the average real estate price is determined for every cell via the polygons generated by Voronoi tessellation (building block 9) [37]. As every cell has an associated real estate price, a price level can be selected for every subscriber’s home and work locations (building block 10). Finally, these indicators are aggregated into a format suitable for modeling (building block 11).

In this study, the normalization technique [38] is performed due to the dynamic range and the parameters’ value differences. This technique can be formulated and performed using Equation (1) for adjusting values measured on different scales to a notionally common parameters’ scale for the ranges from +1 to −1. The final values between +1 and −1 can be generated based on the minimum and maximum input values. Using the normalization technique would significantly reduce the errors raised by differences in the parameter range.

(1) $$x_{N}=\left(\left(\frac{x-X_{\min}}{X_{\max}-X_{\min}}\right)\times 2\right)-1$$

where, $x_{N}$ represents the normalized data in the range of +1 and −1. $X_{min}$ represents the lowest number and $X_{max}$ the highest number in the dataset, respectively.

**Figure 4:**
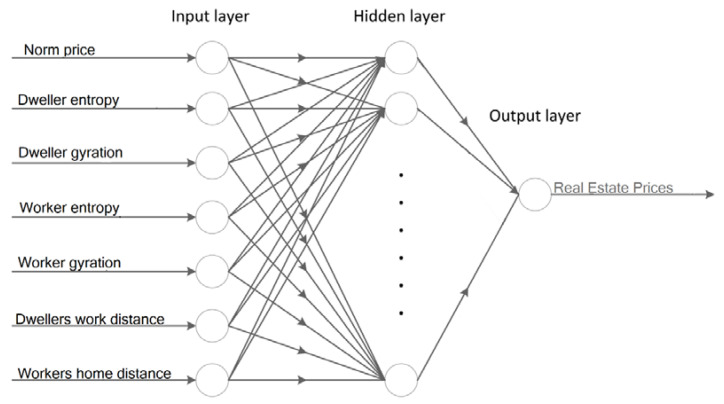
Architecture of the proposed model based on MLP.

This study proposes an efficient classification method based on artificial neural networks [39]. This study’s principal ANN modeling is conducted using a multi-layered perceptron’s machine learning method [40]. A multi-layered perceptron variation of the neural networks works according to the feedforward neural network principle, a standard yet powerful neural network. MLP can efficiently generate the output variables’ values according to the input variables through a non-linear function. MLP, as one of the simplest artificial intelligence methods for supervised learning, consists of several perceptrons or neurons [41]. MLP uses a backpropagation algorithm, which is supervised learning of artificial neural networks using gradient descent. The perceptron models the output according to its weights and the non-linear activation functions. Figure 4 represents an implementation of the model with the detailed architecture and the input variables of the MLP. According to implemented architecture, the model includes three learning phases. The first phase obtains and inserts seven input variables. The next phase, which is devoted to the hidden layers, contains several sets of hidden neurons. The number of neurons in the hidden layer can be modified and tuned to deliver higher performance. In this study, the number of neurons in the hidden layer is an efficient factor in improving model accuracy. The model’s third layer, or so-called output layer, regulates and delivers the output variable, which is the real estate price.

The popularity of MLP has recently been increasing due to its robustness and relatively high performance [42]. Literature includes several comparative studies where MLP models outperform other models [43]. MLP has also shown promising results in modeling a diverse range of data and applications. Therefore, in this study, it had been selected as a suitable modeling algorithm. The essential information and formulation of MLP are described as follows. The output value of f(x) is calculated using Equation (2). According to [39,40,41,42], a hidden layer connects the input layer to the output layer and computes it as follows.

$${f:R}^{I}\to R^{O}$$

(2) $$f\left(x\right){\;=\;K(b}^{\left(2\right)}{+\;w}^{\left(2\right)}\left(Q\left(b^{\left(1\right)}{+\;w}^{\left(1\right)}x\right)\right))$$

where, b and w represent the bias and weights. Furthermore, K and Q denote the activation functions.

In addition, Equation (3) is devoted to representing the hidden layer and is described as follows.

(3) $$h\left(x\right){\;=\;Q(b}^{\left(1\right)}{+\;w}^{\left(1\right)}x)$$

Here, Q’s activation functions are obtained through Equations (4) and (5) as follows.

(4) $$\mathrm{Tanh}\left(x\right){\;=\;(e}^{x}{\;+\;e}^{-x}{)/(e}^{x}\;-{\;e}^{-x})$$

(5) $$\mathrm{Sigmoid}\left(x\right){\;=\;1/(1\;+\;e}^{-x})$$

where Sigmoid(x) delivers a slower response compared to the Tanh(x). In addition, the output vector is formulated and presented according to Equation (6) as follows.

(6) $$o\left(x\right){\;=\;K(b}^{\left(2\right)}{\;+\;w}^{\left(2\right)}h\left(x\right))$$

In MLP, one input layer, one hidden layer, and one output layer for the neural network have been set during training and testing [40]. Furthermore, the basic concepts and problem-solving strategy of particle swarm optimization (PSO) evolutionary algorithm [44] are used to enhance the MLP classifier’s performance [40]. To train the MLP, the advanced evolutionary algorithm of PSO is proposed. When MLP is trained with PSO, the combination is called MLP-PSO, which provides a robust technique to model several non-linear real-life problems [41]. MLP-PSO has recently been used in several scientific and engineering applications with promising results. Comparative analysis of PSO’s performance with other evolutionary algorithms in training neural networks has shown reliable results where PSO in several cases outperforms other algorithms [42,43].

The PSO, as an efficient stochastic based algorithm, works based on finding global optimization. The algorithm follows the population-based search strategy, which starts with a randomly initialized population for individuals. The PSO, through adjusting each individual’s positions, finds the global optimum of the whole population [44]. Each individual is tuned by adjusting the particles’ velocities in the search space for particles’ social and cognition behaviors as follows.

(7) $$V_{i}\;\left(t+1\right)={\;V}_{i}\;\left(t+1\right)+c_{1}\times \mathrm{rand}\left(1\right)\times \left({\mathrm{lbest}}_{i}-X_{i}\right)+c_{2}\times \mathrm{rand}\left(1\right)\times \left({\mathrm{gbest}}_{i}-X_{i}\right)$$

(8) $$X_{i}\;\left(t+1\right)={\;X}_{i}\;\left(t\right)+V_{i}\;\left(t+1\right)$$

where *rand*(1) is a random function for producing values between 0 and 1. Furthermore, $c_{1}$ and $c_{2}$ remain constants with values between 0 and 2. In this study, $c_{1}$ and $c_{2}$ are set to 2 throughout the modeling [40]. The algorithm starts by initializing $V_{i}\;\left(t\right)$ and $X_{i}\;\left(t\right)$ which represents the population of particles and velocity, respectively [34]. In the next step, the fitness of each particle is calculated. Further, $\left(lbest_{i}\right)$ computes the local optimum through elevating the fitness of particles in every generation. $\left(gbest_{i}\right)$ identifies the particle with better fitness as the global optimum. The $V_{i}\;\left(t+1\right)$ delivers the new velocity and $X_{i}\;\left(t+1\right)$ is generating the new positions of the particles. The algorithm is adjusted to reach the maximum iteration of the velocity range [41]. The modeling includes two phases of training and testing. Additionally, 70% of the data is used for training and 30% for testing. Furthermore, the evaluation of the performance of the models was performed by the use of correlation coefficient (CC), scattered index (SI), and Willmott’s index (WI) of agreement, Equations (3)–(5) [42,43].

(9) $$\mathrm{CC}=\frac{\left(\sum_{i=1}^{n}O_{i}P_{i}-\frac{1}{n}\sum_{i=1}^{n}O_{i}\sum_{i=1}^{n}P_{i}\right)}{\left(\sum_{i=1}^{n}O_{i}{}^{2}-\frac{1}{n}{\left(\sum_{i=1}^{n}O_{i}\right)}^{2}\right)\left(\sum_{i=1}^{n}P_{i}{}^{2}-\frac{1}{n}{\left(\sum_{i=1}^{n}P_{i}\right)}^{2}\right)}$$

(10) $$\mathrm{SI}=\frac{\sqrt{\frac{1}{n}\sum_{i=1}^{n}{\left(P_{i}-O_{i}\right)}^{2}}}{\bar{O}}$$

(11) $$\mathrm{WI}=1-\left(\frac{\sum_{i=1}^{n}{\left(O_{i}-P_{i}\right)}^{2}}{\sum_{i=1}^{n}{\left(\left|P_{i}-{\overset{\_\_}{O}}_{i}\right|+\left|O_{i}-{\overset{\_\_}{O}}_{i}\right|\right)}^{2}}\right)$$

where, *O* refers to the output value, *P* refers to the predicted value, and *n* refers to the number of data [43].

## 3. Results

The results and further description of statistical modeling, training, and testing are presented as follows.

### 3.1. Statistical Results

Statistical analysis is conducted by SPSS software V. 22 using ANOVA analysis [45]. Table 1 includes the sum of squares, mean square, F value, and significance index between groups. According to Table 1, all the variables which have been selected as the independent variables have significant effects on the real estate price as the only dependent variable.

### 3.2. Training Results

Using three performance indexes, namely, MSE, SI, and WI, Table 2 summarizes MLP and MLP-PSO models’ training results. The number of the neurons are 10, 12, and 14, and the population sizes vary from 100, 150, to 200.

### 3.3. Testing Results

**Figure 5:**
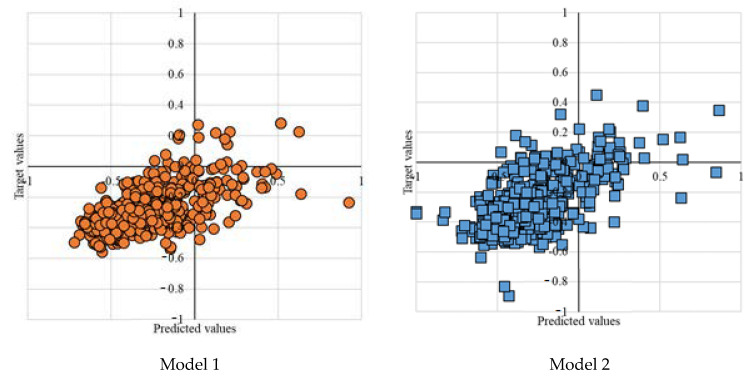

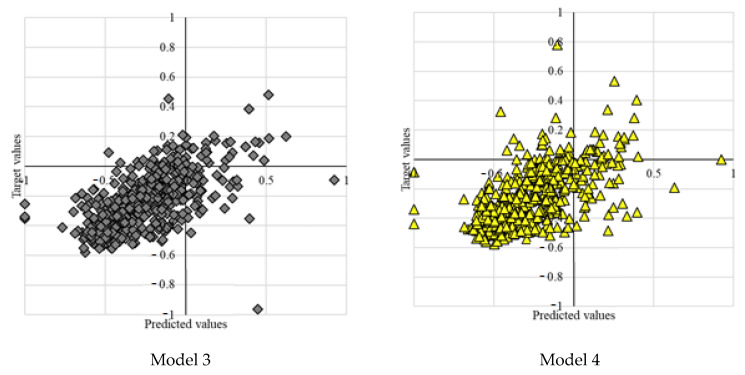
Plot diagrams for the selected models.

**Figure 6:**
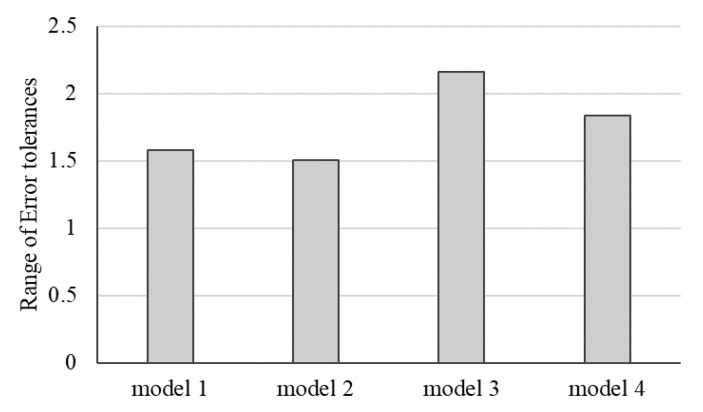
The range of error tolerances for the testing results.

Four models with various neuron numbers and population sizes in Table 3 represent the experimental results. The MLP-PSO with ten neurons in the hidden layer and population size of 100 outperforms other configurations. Figure 5 further presents the plot diagrams of the models. Studying the range of error tolerances of the models for the testing results is also essential to identify the model with higher performance. Figure 6 visualizes the models’ error tolerances.

Model 1 represents a single MLP with ten neurons in the hidden layer. Model 2 is an MLP-PSO with ten neurons in the hidden layer and population size of 100. Model 3 represents an MLP-PSO with 12 neurons in the hidden layer and population size of 100. Model 4 is an MLP-PSO with 14 neurons in the hidden layer and population size of 150.

Performance evaluation of the four proposed models for the testing phase indicates that hybrid model 2 with fewer neurons in the hidden layer and lower population size outperforms other models. As illustrated in Figure 6, of the four models’ range of error tolerances, model 2 shows promising results.

### 3.4. The Interactions of Variables on the Testing Results

Analyzing the outputs of the testing phase for studying the effect of each independent variable on the real estate price indicated that real estate price has an indirect relation with dweller gyration, dweller entropy, workers’ gyration, and workers’ home distance and has a direct relation on workers’ entropy and dwellers’ work distances. It can be claimed that, according to the observations, working and flow of activities and entropy of mobility are from areas with lower real estate prices to areas with higher real estate prices.

**Figure 7:**
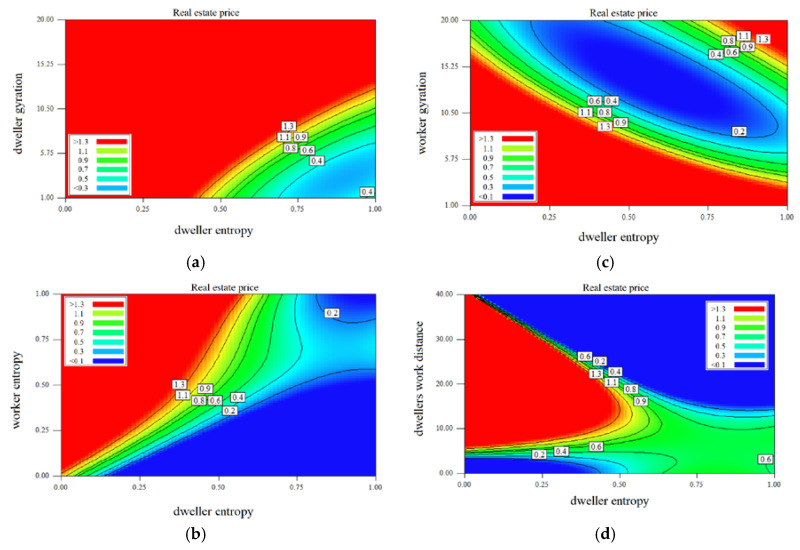

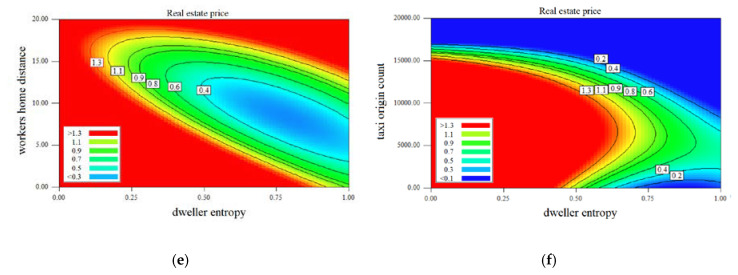
Illustration of the effect of independent variables on real estate price: (**a**) dweller gyration, (**b**) worker entropy, (**c**) worker gyration, (**d**) dwellers work distance, (**e**) workers home distance, and (**f**) taxi origin count.

Figure 7a represents the normalized property process’s dependence on entropy and gyration of inhabitants living in Budapest. The contour lines on the heat map chart showing the levels of property prices suggest that there is a strong influence of property prices on entropy and gyration of the dwellers. Additionally, entropy and gyration show a linear relationship with the home’s prices. The higher the gyration beside the same value of entropy, the higher the property price is. On the other hand, it seems that people have the same level of gyration, but higher entropy (visiting more places) stay in poorer zones in the city. The right bottom part of the heat map chart shows the lower property price domain. The people staying in cheaper homes visit many city locations, therefore having entropy higher than 0.7 and having a radius of gyration less than 6 km.

Figure 7b illustrates the distribution of housing prices as functions of the average of entropy in cells where phone users are working and where people have their homes. The straight line between the bottom left and the top right corner covers the cells where the entropy (level of visiting diversity) is similar. The low entropy in working and after work hours is typical in the cells of wealthy areas. The higher both the entropies in a cell lower the price of the properties. This chart highlights the economic status of locations depending on the visiting habit of the dwellers. The cells where the workers represent the middle and high range entropy group (>0.75), and the inhabitants’ activity is in the middle range (0.4–0.6) belong to the expensive neighborhood (left upper part of the chart). These cells are located in the most upbeat working region (financial district) of the city. The housing prices are proportionally lower at places where the most diverse visiting behavior population works and lives by the increasing entropy of home cells. The area where the high entropy dwellers live and only limited entropy people work seems to be relatively cheap. In these zones, limited job opportunities are available and the inhabitants have to visit several locations on a weekly basis.

The gyration of the area where people are working and the entropy of the same locations are used as home are the places that have a remarkable interrelation to housing prices (Figure 7c). The most expensive properties can be found in the cells where the inhabitants visit only a few locations (entropy is <0.25), and the gyration of the workers is relatively high (>10 km). The higher the gyration level in the working place cell, the lower the housing price if the home cell entropy is in the middle range (0.4–0.6). The dwellers visit the same level of destinations but have a bigger radius of gyration living in cheaper neighborhoods. In the region where the inhabitants’ entropy is relatively high (>0.75) lower housing prices belong to higher worker gyration until its value is lower than 10 km. However, the properties are more expensive if the gyration is bigger than 15 km.

The level of gyration in a cell is significantly correlated with the distance between the workplace and the dwellers’ home locations. The people living far from their job locations have to spend more time traveling. Therefore, their opportunities to visit several places in the city are limited. This observation is confirmed in Figure 7d. It predicted coherency of the home and work locations’ cell level distances and dwellers entropy and the housing prices. There are no properties available at the regions where the home-work distance and entropy are small (left bottom corner of the heat map) or both of them are high (top right corner of the chart). The home’s prices increase with the higher the distance between home and workplaces, the higher home’s prices in the cells having a value of entropy below 0.5. It seems that people visiting only a few locations (i.e., home, work, school, etc.) could afford to live in more expensive districts and travel more for their work. These significant characteristics are not typical in the cells having higher diversity of visited locations. The people living in middle price (0.6–0.8 million HUF) homes have higher entropy and are ready to travel long distances for their jobs.

Figure 7e illustrates how housing prices can be estimated by taking into account the mean home-work approach and entropy in the cells. The cheapest flats can be found in those cells where the inhabitants have diversified visiting habits and people having their workplaces within 5–10 km from home. The houses are proportionally more expensive by the difference of this home-work distance range. It is also interesting that on the same level of home-work mileage, the property prices in cells are higher where the mean entropy is smaller. The explanation for this trend could be that the more expensive neighborhood has more easily accessible services and facilities, and the dwellers need to visit fewer places.

## 4. Conclusions

Call detail records with mobility information help telecommunication companies map the users’ accurate locations and entropy activities for analyzing social, economic, and related capabilities in the subset of the smart cities category. The lack of an exact solution to transform the data into practical tools for better understanding the nature of the effect of telecommunication technologies in today’s life leads researchers to use some additional and useful tools for making a user-friendly system under telecommunication technologies like machine learning tools. The present study develops single and hybrid machine learning techniques to analyze and estimate estate prices according to the call data records, including mobility entropy factors. These factors include dweller entropy, dweller gyration, worker entropy, worker gyration, dwellers’ work distance, and workers’ home distance. Modeling had performed using the machine learning method of multi-layered perceptron trained with the evolutionary algorithm of particle swarm optimization for optimum performance. Results have been evaluated by mean square error, sustainability index, and Willmott’s index. Statistical analysis indicated that all the selected independent variables have a significant effect on the dependent variable. According to the results, the hybrid ML method could successfully cope with estimating the estate price with high accuracy over the single ML method. Analyzing the outputs of the testing phase for studying the effect of each independent variable on the real estate price indicated that real estate price has an indirect relation with dweller gyration, dweller entropy, workers’ gyration, and workers’ home distance and have a direct relation with workers’ entropy, and dwellers’ work distance. It can be claimed that, according to the observations, working and flow of activities and entropy of mobility are from areas with lower estate prices to regions with higher estate prices.

For future research, exploring other cities of the country using the proposed model is encouraged. In addition, developing more sophisticated machine learning models to study the CDR data with higher performance is suggested. The future of the research on CDR data with machine learning will not be limited to real estate price prediction. Further research on mobility modeling would be beneficial in a wide range of applications, for example, COVID-19 outbreak and its governance modeling.

## Figures and Tables

**Table 1:** The statistical analysis of dependent and independent variables.

			Sum of Squares	Mean Square	F	Sig.
dweller entropy × estate price	Between Groups	(Combined)	83.073	0.044	1.558	0.000
		Linearity	0.871	0.871	30.899	0.000
		Deviation from Linearity	82.201	0.043	1.542	0.000
dweller gyration × real estate price	Between Groups	(Combined)	98.941	2256	0.044	0.000
		Linearity	6.146	1	6.146	0.000
		Deviation from Linearity	92.795	2255	0.041	0.000
Worker entropy × real estate price	Between Groups	(Combined)	86.504	1867	0.046	0.000
		Linearity	4.592	1	4.592	0.000
		Deviation from Linearity	81.912	1866	0.044	0.000
Worker gyration × real estate price	Between Groups	(Combined)	98.704	2262	0.044	0.000
		Linearity	0.156	1	0.156	0.000
		Deviation from Linearity	98.548	2261	0.044	0.000
Dwellers work distance × real estate price	Between Groups	(Combined)	98.168	2234	0.044	0.000
		Linearity	2.306	1	2.306	0.000
		Deviation from Linearity	95.862	2233	0.043	0.000
Workers home distance × real estate price	Between Groups	(Combined)	99.112	2261	0.044	0.000
		Linearity	3.506	1	3.506	0.000
		Deviation from Linearity	95.605	2260	0.042	0.000

**Table 2:** Evaluation of the performance of the models for the training phase.

Performance Index	Neuron Number	10	12	14	Pop. size
MSE	MLP	0.0419	0.0427	0.0424	-
	MLP-PSO	0.0301	0.03	0.0407	100
		0.042	0.0397	0.029	150
		0.0406	0.0391	0.0395	200
SI	MLP	−0.15585	−0.15818	−0.15873	-
	MLP-PSO	−0.11380	−0.11021	−0.15175	100
		−0.15610	−0.14916	−0.10855	150
		−0.15234	−0.14580	−0.14714	200
WI	MLP	0.70706	0.70372	0.71219	-
	MLP-PSO	0.82790	0.82585	0.71817	100
		0.71428	0.72410	0.83918	150
		0.72033	0.74003	0.73372	200

**Table 3:** Evaluation of the performance of the models for the testing phase.

Model		MSE	SI	WI
Model 1	MLP 10	0.0403	−0.14857	0.70780
Model 2	MLP-PSO 10-100	0.0393	−0.14723	0.77701
Model 3	MLP-PSO 12-100	0.0411	−0.17166	0.78043
Model 4	MLP-PSO 14-150	0.0414	−0.15900	0.76584
